# Erythropoiesis Suppression Is Associated with Anthrax Lethal Toxin-Mediated Pathogenic Progression

**DOI:** 10.1371/journal.pone.0071718

**Published:** 2013-08-19

**Authors:** Hsin-Hou Chang, Tsung-Pao Wang, Po-Kong Chen, Yo-Yin Lin, Chih-Hsien Liao, Ting-Kai Lin, Ya-Wen Chiang, Wen-Bin Lin, Chih-Yu Chiang, Jyh-Hwa Kau, Hsin-Hsien Huang, Hui-Ling Hsu, Chi-Yuan Liao, Der-Shan Sun

**Affiliations:** 1 Department of Molecular Biology and Human Genetics, Tzu-Chi University, Hualien, Taiwan; 2 Institute of Medical Sciences, Tzu-Chi University, Hualien, Taiwan; 3 Department of Microbiology and Immunology, National Defense Medical Center, Taipei, Taiwan; 4 Institute of Preventive Medicine, National Defense Medical Center, Taipei, Taiwan; 5 Department of Obstetrics and Gynecology, Mennonite Christian HospitalHualien, Taiwan; University of Pittsburgh, United States of America

## Abstract

Anthrax is a disease caused by the bacterium *Bacillus anthracis*, which results in high mortality in animals and humans. Although some of the mechanisms are already known such as asphyxia, extensive knowledge of molecular pathogenesis of this disease is deficient and remains to be further investigated. Lethal toxin (LT) is a major virulence factor of *B. anthracis* and a specific inhibitor/protease of mitogen-activated protein kinase kinases (MAPKKs). Anthrax LT causes lethality and induces certain anthrax-like symptoms, such as anemia and hypoxia, in experimental mice. Mitogen-activated protein kinases (MAPKs) are the downstream pathways of MAPKKs, and are important for erythropoiesis. This prompted us to hypothesize that anemia and hypoxia may in part be exacerbated by erythropoietic dysfunction. As revealed by colony-forming cell assays in this study, LT challenges significantly reduced mouse erythroid progenitor cells. In addition, in a proteolytic activity-dependent manner, LT suppressed cell survival and differentiation of cord blood CD34^+^-derived erythroblasts *in vitro*. Suppression of cell numbers and the percentage of erythroblasts in the bone marrow were detected in LT-challenged C57BL/6J mice. In contrast, erythropoiesis was provoked through treatments of erythropoietin, significantly ameliorating the anemia and reducing the mortality of LT-treated mice. These data suggested that suppressed erythropoiesis is part of the pathophysiology of LT-mediated intoxication. Because specific treatments to overcome LT-mediated pathogenesis are still lacking, these efforts may help the development of effective treatments against anthrax.

## Introduction

*Bacillus anthracis*, the etiological agent responsible for anthrax, is a Gram-positive, nonmotile, aerobic, spore-forming rod-shaped bacterium [1]. Anthrax is a disease primarily affecting herbivores (e.g., cattle, sheep, and goats), which become infected by ingesting contaminated vegetation, water, or soil; humans are generally incidental hosts [1]. Based on the entrance route of *B. anthracis* endospores, 3 types of human manifestations exist: cutaneous, gastrointestinal, and inhalational infections; these can develop into systemic infections with symptoms of hypotension, hemorrhage, multi-organ failure, and sudden shock [2]–[4]. Anthrax lethal toxin (LT) is a major virulence factor of *B. anthracis*, which is composed of 2 polypeptides: the protective antigen (PA, 83 kDa) and the lethal factor (LF, 90 kDa) [5]–[7]. LF is a zinc-dependent metalloprotease, which cleaves the N-terminal domain of mitogen-activated protein kinase kinases (MKKs/MEKs) [8], [9], disrupting 3 downstream mitogen-activated protein kinase (MAPK) pathways: the extracellular signal-regulated kinase (ERK), p38, and the c-Jun N-terminal kinase (JNK) pathways [10], [11]. LF is toxic only when composed with PA to form LT. After binding to one of 2 known cellular receptors, tumor endothelium marker-8 (TEM8) or capillary morphogenesis protein-2 (CMG2) [12], PA is cleaved using a cellular furin-like protease, oligomerized, and used to deliver LF into cells [6], [13]. LT could cause high mortality in experimental mice and rats [14], [15]; however, the pathogenic mechanism of LT that leads to animal death is not well understood. Previous studies have indicated that LT treatments induce TNF-α-independent hypoxia and shock [15]. Because systemic hypoxic symptoms cause animal deaths [15], LT-induced anemia may be involved. Anthrax-mediated hemolytic anemia has been reported in several clinical studies [16]–[18]. An LT-induced hemolysis of human blood was also demonstrated *in vitro* [19]; however, its impact has not been clarified *in vivo*. Because LF can disrupt all 3 MAPK pathways [8], and that the ERK [20], [21], JNK [22], and p38 [23] pathways are all critical to erythropoiesis, we hypothesized that LT may block erythropoiesis and further exacerbate the anemic symptoms. This study clarified the potential involvements of hemolytic anemia and suppressed erythropoiesis in LT-treated C57BL/6J mice. In addition, because a significant induction of serum erythropoietin (EPO) is associated with extensive systemic hypoxia after LT administration in mice [15], we hypothesized that the EPO release may be a self-rescue response. We also evaluated whether enhancing erythropoiesis using EPO treatments may ameliorate LT-induced anemia, and thus, reduce the mortality of LT-treated mice.

## Results

### Lethal Toxins Induced Hemolysis in Mice

**Figure 1:**
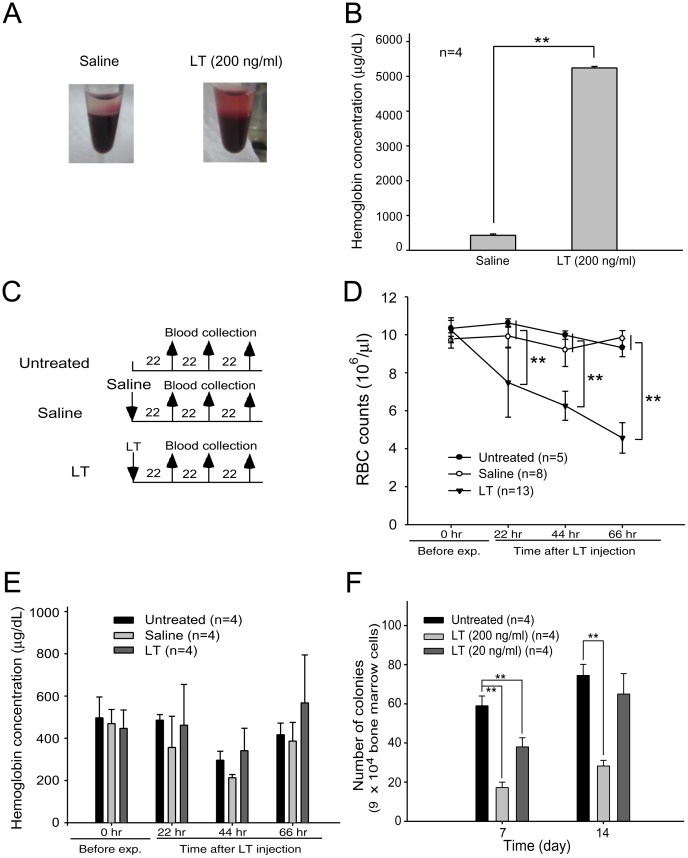
Effects of LT on hemolysis and *in vitro* erythroid colony-forming cell assay. An image of LT (200 ng/ml)-induced hemolysis *in vitro* was compared with a saline-treated control group (A). After peripheral blood cells were incubated with or without LT at 37°C for 2 hours, plasma hemoglobin levels (µg/dL) were measured using Drabkin’s reagent (B). The experimental outline of the *in vivo* hemolysis assay is shown, in which untreated and saline-treated groups served as negative control groups (C). The RBC counts (D) and the levels of cell-free plasma hemoglobin (E) were determined 22, 44, and 66 hours after the mice were treated with LT. The *in vitro* erythroid colony-forming cell assay was performed by incubating murine BM cells with or without LT (200 ng/ml or 20 ng/ml). Colonies of progenitor cells were counted on Days 7 and 14 post-erythroid differentiation initiation (F). Untreated BM cells were used as a control. ** *p*<0.01, comparisons between groups are indicated. Data are reported as mean ± SD.

Following previous studies using human blood [19], we reproduced *in vitro* LT-mediated hemolysis experiments using mouse blood. We found that LT treatments (200 ng/ml) had a lesser effect in mice (Figure 1A and 1B) compared to humans ([19] and authors’ unpublished results). *In vivo* analyses indicated that LT treatments gradually reduced RBC counts of mice prior to death (Figure 1C, experiment outline, and Figure 1D). When hemolysis occurs within the blood circulation, hemoglobin is normally cleared by the hemoglobin-scavenging mechanisms [24], [25]. When a massive hemolysis is severe enough to saturate the hemoglobin-scavenging mechanism capacity, the plasma level of the cell-free hemoglobin increases [26]. To investigate the level of hemolysis in LT-mediated anemia, plasma hemoglobin levels of LT-treated (1.5 mg/kg; a lethal dose) mice were measured. Compared to the untreated and saline-treated groups, the level of plasma hemoglobin did not increase significantly after LT treatments (Figure 1C, experiment outline, and Figure 1E). Based on these results, LT-induced hemolysis might not be the only reason low RBC counts were induced. Because LT suppresses all 3 MAPK pathways [8], and that ERK [20], [21], JNK [22], and p38 [23] are critical to erythropoiesis, we hypothesized that the production and the maturation of the RBC (i.e., erythropoiesis) might be affected by LT. To ascertain whether LT could influence erythropoiesis of primary erythroid precursor cells, isolated mouse BMs were treated with an erythrocytic differentiation-inducing cocktail that contained native RBC synthesis-promoting cytokine EPO, and then treated with or without LT. Erythroid progenitor cells [i.e., burst-forming unit-erythroid (BFU-Es) colonies] appeared after 7 to 14 days in untreated groups, whereas the colonies were significantly suppressed in LT-treated groups, especially at the high dose (Figure 1F, for 200 ng/ml groups). These results suggest that LT is able to block erythropoiesis of primary erythroid precursor cells.

### Apoptosis is Involved in LT-catalytic Activity-dependent Erythropoiesis Suppression

**Figure 2:**
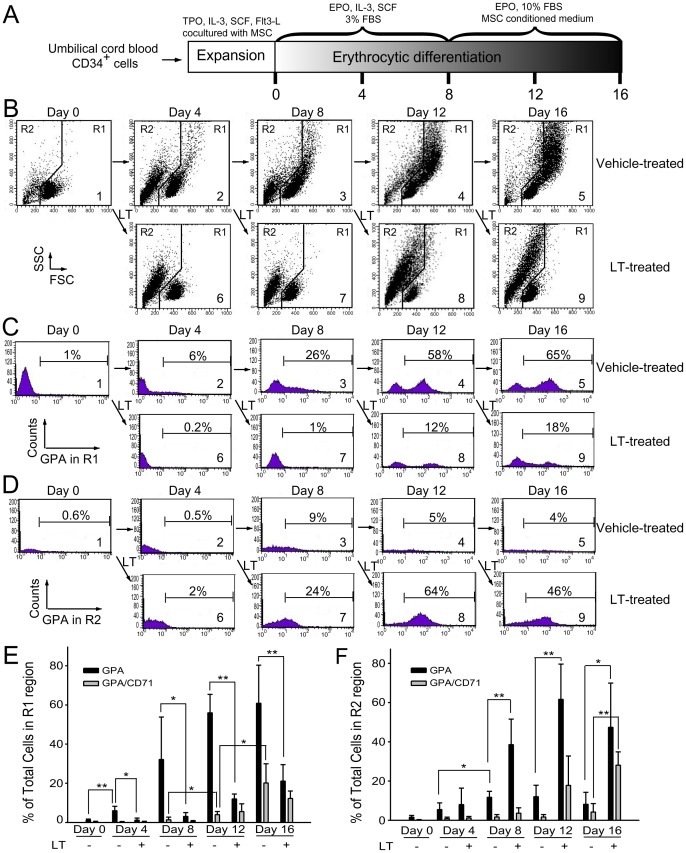
Suppressive effect of LT on *in vitro* erythrocytic differentiation. The experiment outline of *in vitro* erythrocytic differentiation using human cord blood-derived CD34^+^ cells is shown (A). Flow cytometry analysis of the cell size (FSC) and cell granularity (SSC) are shown at various time points (B). The percentage of GPA^+^ (erythrocytic marker) cells in the R1 and R2 regions are shown in (C) and (D), respectively. The percentage of GPA and GPA/CD71 expression cells in the R1 and R2 regions at various differentiation times are shown in (E) and (F), respectively. Cells were treated with LT (20 ng/ml) during differentiation on Days 0, 4, 8, and 12. After the 4-day LT treatments, cells in each differentiation stage were harvested for additional flow cytometry analysis. Representative cell-populations and histograms (B–D) were shown. The total cell number was defined as 100%. *p<0.05, ** *p*<0.01, comparisons between groups are indicated. Data are reported as mean ± SD and represent results from 4 independent experiments.

**Figure 3:**
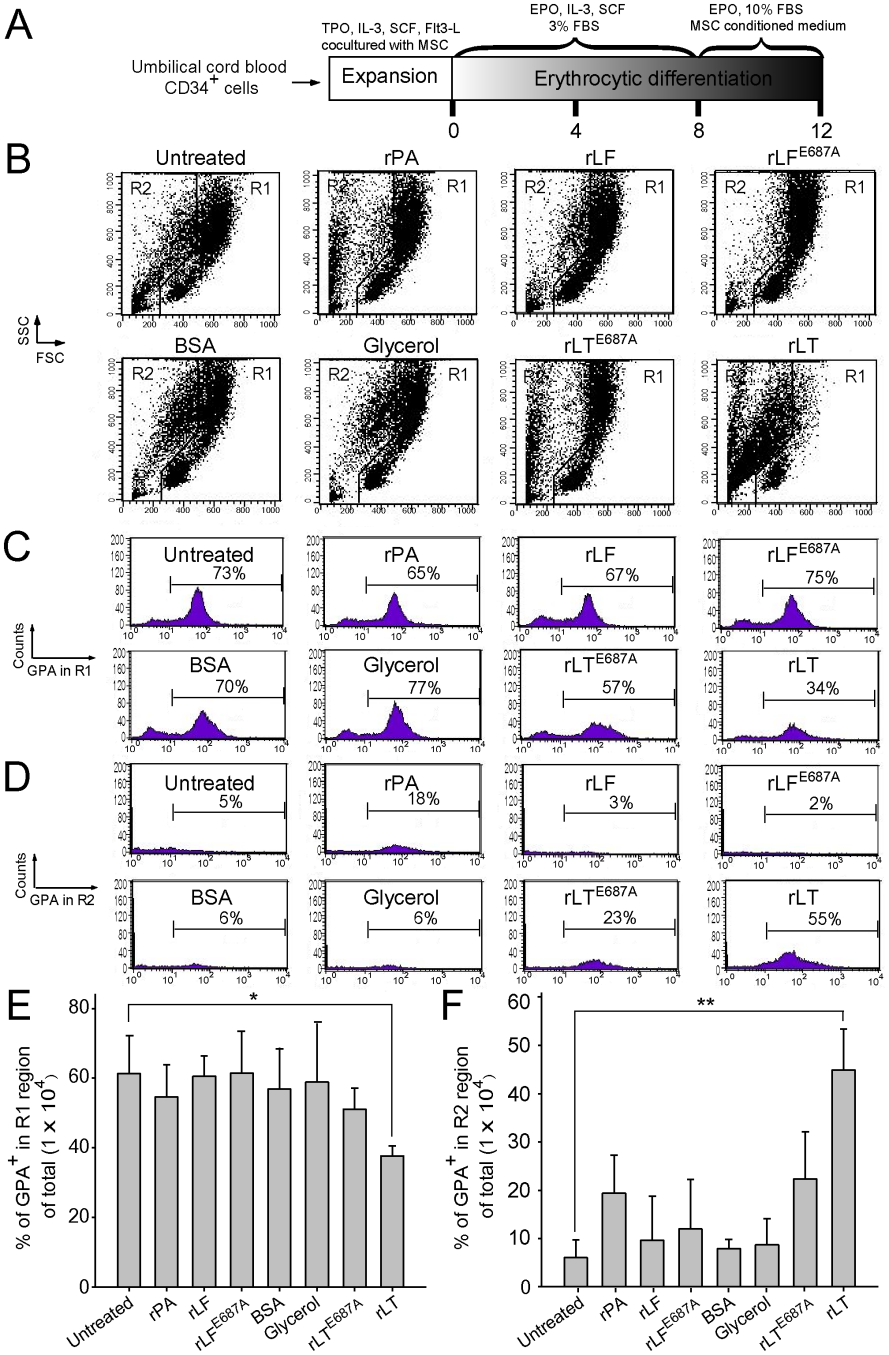
Proteolytic activity of LT component lethal factor is essential for the suppression of erythrocytic differentiation. The timetable of *in vitro* erythrocytic differentiation using human cord blood-derived CD34^+^ cells is shown (A). To address the effect with or without proteolytic activity, rLF and rLF^E687A^ were employed, respectively. Flow cytometry analysis of the cell size (FSC) and cell granularity (SSC) are shown at various times (B). The percentage of GPA^+^ (erythrocytic marker) cells in the R1 and R2 regions are shown in (C) and (D), respectively. The percentage of GPA expression cells in the total cells (1×10^4^) in the R1 and R2 regions for different treatments are shown in (E) and (F), respectively. LT and control proteins such as rPA (5 µg/ml), rLF (5 µg/ml), rLF^E687A^ (5 µg/ml), rLT (5 µg/ml rPA +5 µg/ml rLF), and rLT^E687A^ (5 µg/ml rPA +5 µg/ml rLF^E687A^) were added into the medium by differentiation on Day 8. The cells were then analyzed using flowcytometry 4 days later. Groups treated with or without a (untreated) vehicle and BSA (10 µg/ml) served as negative controls. Representative cell-populations and histograms (B–D) were shown. Total cell number was defined as 100%. *p<0.05, ** *p*<0.01, comparisons with untreated groups are indicated. Data are reported as mean ± SD and represent results from 4 independent experiments.

**Figure 4:**
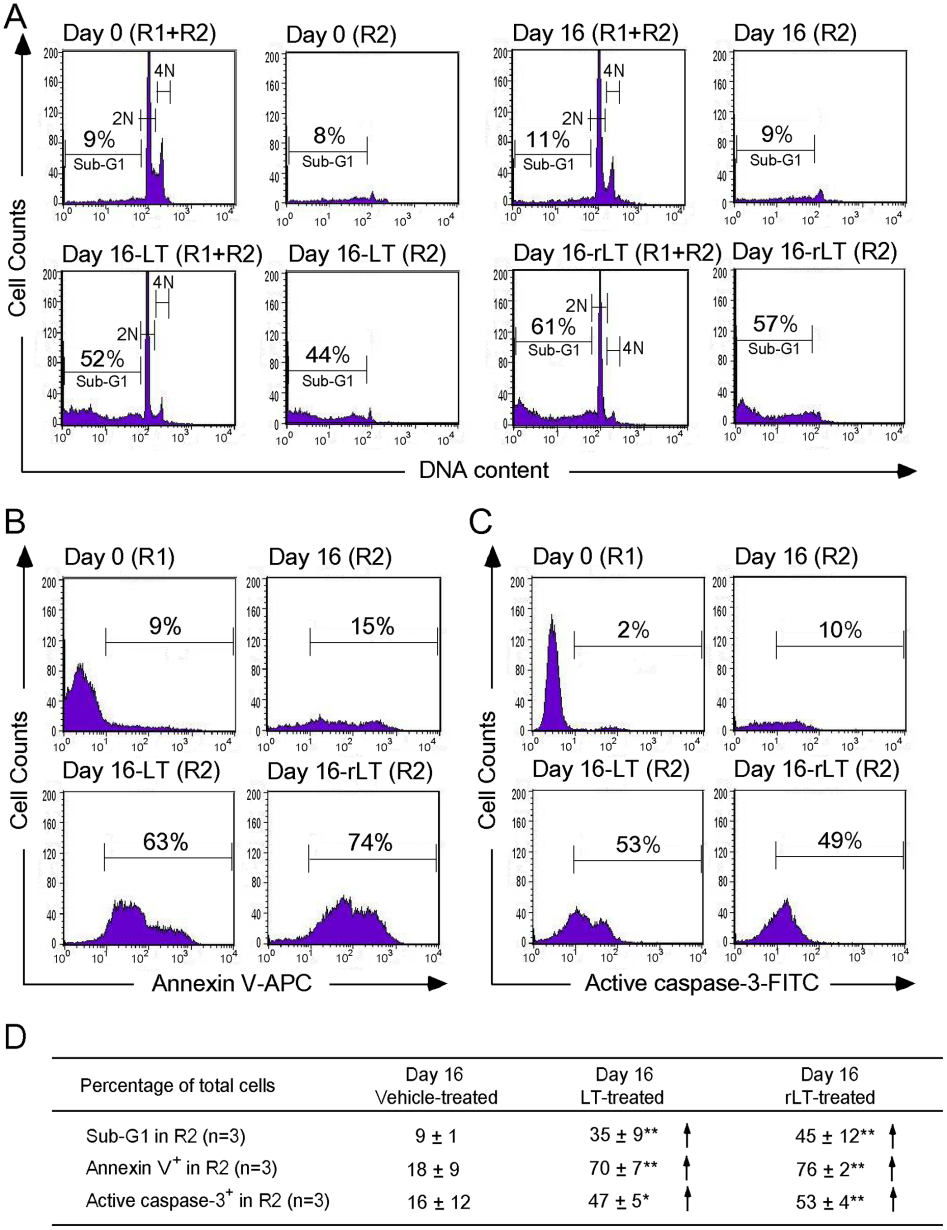
Characterizations of LT-induced hypoploid cells. Human cord blood-derived CD34^+^ cells were treated using LT and rLT on Day 12 and analyzed on Day 16 during the *in vitro* differentiation of erythrocytes. The DNA contents were revealed by fluorescence intensities of propidium iodine (PI) staining. The hypoploid cells were increased in LT-treated and rLT-treated groups (A). Annexin V-APC (B) and active caspase-3-FITC antibodies (C) were used to characterize the apoptotic cells in R2 region by flow cytometry. The percentage of sub-G1 cells on day 0 and annexin V^+^ and active caspase-3^+^ cells of R1 region on day 0 were used as basal level controls. Representative histograms (A–C) and summarized events (D) were shown. *p<0.05, ** *p*<0.01, compared to vehicle-treated controls. Data are reported as mean ± SD and represent results from 3 independent experiments.

To investigate the suppression mechanism on erythropoiesis, *in vitro* erythroid differentiation of cord blood-derived CD34^+^ hematopoietic stem cells (HSC) in a 16 day course was analyzed. To determine the effects of LT on various differentiation stages, cells were divided into 9 groups [Figure 2B (1–9), 2C (1–9), and 2D (1–9)], and then subjected to vehicle [diluents: cell culture medium; Figure 2B (1–5), 2C (1–5), and 2D (1–5)] and LT [Figure 2B (6–9), 2C (6–9), and 2D (6–9)] groups. Cells were treated with vehicle or LT on Days 0, 4, 8, and 12 (Figure 2B–2D, 2–5 vehicle groups, 6–9 LT groups). Toxin treatments were conducted for 4 days for each of LT-treated groups (Figure 2B–2D: Group 6, Days 0–4; Group 7, Days 4–8; Group 8, Days 8–12; Group 9, Days 12–16). After treatments, surface markers of erythrocytic progenitor cells, such as CD235a (glycophorin A, GPA) and CD71 (the transferrin receptor), were examined using flow cytometry. CD71 is expressed by a wide variety of hematopoietic cells, including BFU-Es, CFU-Es, and proerythroblasts, but it is not present on mature erythrocytes [27], whereas GPA is a late erythroid marker expressed on erythroblast cells and mature erythrocytes, but not on the earliest precursor cells [28]. The data revealed that cell size (FSC), cell granularity (SSC), and the percentage of GPA^+^ and GPA^+^/CD71^+^ cells were gradually increased in the R1 region during differentiation (Figure 2A, experiment outline, Figure 2B, 2C, and 2E). In contrast, the populations of erythrocytic progenitor cells (Figure 2C–2F, GPA^+^ and GPA^+^/CD71^+^) were gradually shifted from the R1 (Figure 2B, larger cells) to the R2 region (Figure 2B, smaller cells) after LT treatments at varied differentiation times (Figure 2B–2F; E, F, * *p*<0.05, ** *p*<0.01). To further explore the enzymatic activity requirement of LT on erythropoiesis suppression, recombinant PA (rPA), and recombinant LF (rLF), and E687A mutant LF (rLF^E687A^) [which form recombinant wild-type LT (rLT) and catalytic mutant LT (rLT^E687A^), respectively] were produced and purified [29]. In agreement with the treatments using LT, rLT induced a similar cellular response that shifted the populations of the erythrocytic progenitor cells from the R1 to the R2 region, compared to the untreated, vehicle, and other recombinant protein control groups (Figure 2B–2F vs. Figure 3B–3F). The catalytic mutant LT (rLT^E687A^) did not have such effects (Figure 3B–3F). Because sub-G1 dead cells are smaller than normal cells, propidium iodine (PI) staining was further used to verify whether cell death was involved. In a representative PI-staining analysis, LT and recombinant LT (rLT) elicited 52% and 61% of sub-G1 death cells, respectively [Figure 4A, Day 16-LT (R1+R2) groups and Day 16-rLT (R1+R2) groups vs. untreated control groups Day 0 (R1+R2) with only 9% sub-G1 and Day 16 (R1+R2) only 11% sub-G1 cells]. Among these 52% [Day 16-LT (R1+R2) groups] and 61% [Day 16-rLT (R1+R2)] sub-G1 cells, 44% and 57% of which belong to R2 population [Figure 4A, Day 16-LT (R2), and Day 16-rLT (R2) groups], while only 8% and 4% belong to R1 population (data not shown) (aforementioned Figure 4A results are from one representative experiment, averaged results are showed in Figure 4D). This indicates that the major populations of cell in R2 region are actually dead after LT treatments (92% and 95% of R2 region; data not shown). We further used annexin V and active casepase-3 antibody to characterize whether these are apoptotic cells. Our data indicates that after LT and rLT treatments, 70% to 76% of total cells (weighting 94% and 95% of R2 population) became annexin V positive, and 47% to 53% of total cells became active-caspase-3 positive cells (Figure 4D). This indicates that those LT-elicited hypoploid cells in R2 region are apoptotic cells. Our unpublished data also indicate that there are approximately 80% of R2-region cells expressing erythroid markers GPA on their surfaces. At the same time, about 61% GPA^+^ cells are annexin V positive. To verify whether LT preferentially kills those differentiated cells, we performed LT-treatments of expanded CD34^+^ cells (4-day treatment, a same time course of aforementioned differentiating cells) for comparisons. Intriguingly, we did not observe significant elicitation of apoptotic cells in R2 region (our unpublished data). These results suggest that LT can’t kill undifferentiated CD34^+^ precursor cells. Our data collectively suggest that LT preferentially kills GPA^+^-differentiating erythroid precursor cells but not GPA^−^, CD34^+^ progenitors. As a result, our data collectively suggest that anthrax LT can suppress erythropoiesis in part by killing the erythroid progenitors through induction of caspase-3 dependent apoptosis. In addition, this suppression is dependent on the catalytic activity of LT.

**Figure 5:**
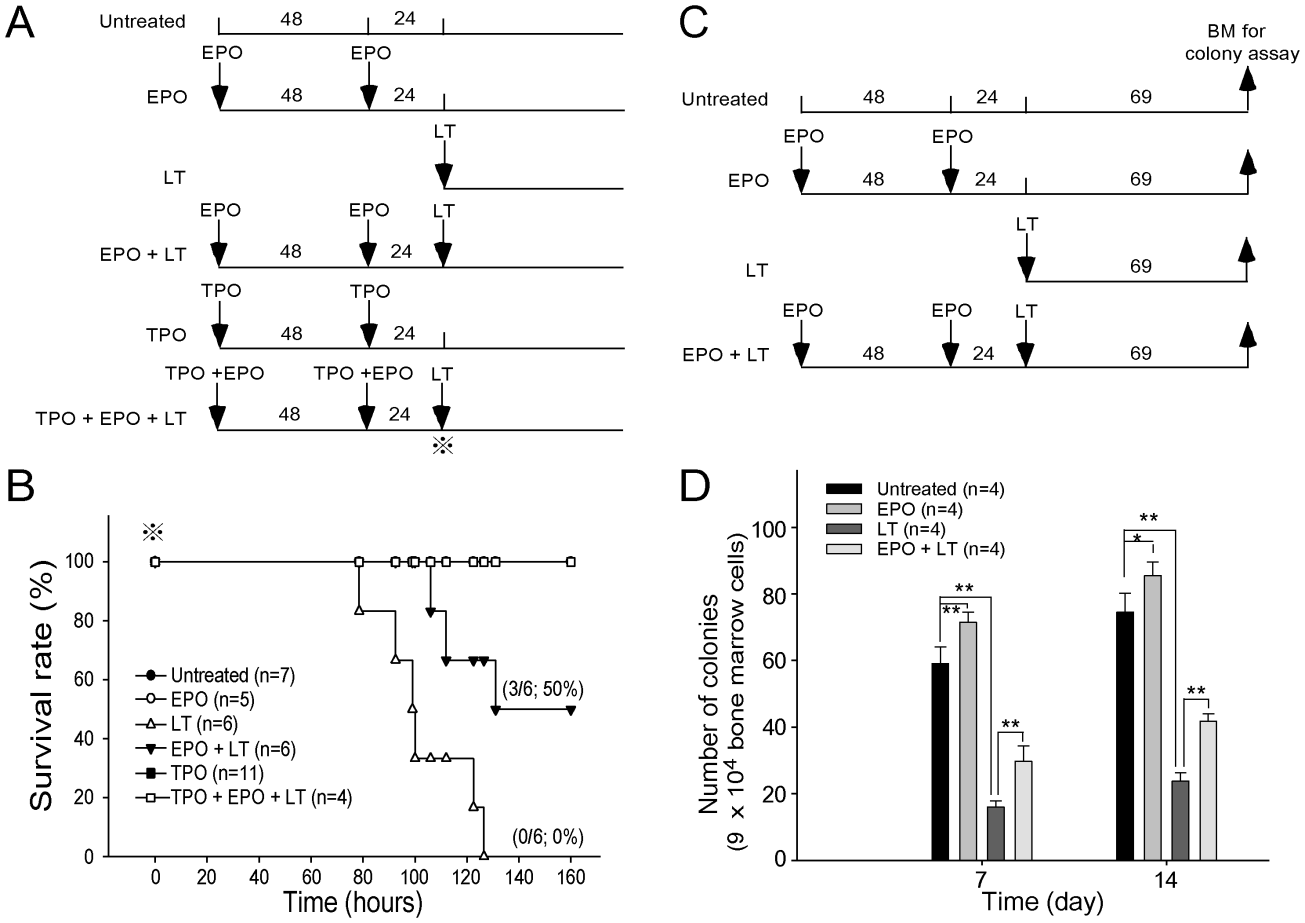
EPO treatments reduced LT-mediated mortality and erythropoiesis suppression in mice. The experimental outline of the survival rate analysis is shown in (A). The survival rate of mice treated with EPO, LT, TPO, EPO in addition LT or TPO, and EPO plus LT is shown in (B). Untreated mice were used as negative controls. The symbol ⋇ marks in (A) and (B) indicate the starting point for recording mortality. The experimental outline of the *in vivo* erythroid colony-forming cell assay is shown in (C). Colony numbers of BM cells from mice treated with EPO, LT, or EPO in addition to LT by the erythroid colony-forming cells assay on Days 7 and 14 is shown in (D). BM cells from untreated mice were used as controls. *p<0.05, ** *p*<0.01, comparison between groups are indicated. Data are reported as mean ± SD, and represent results from 4 independent experiments.

### Erythropoietin Treatments Reduced LT-mediated Mmortality, Aanemia, and Erythropoiesis Suppression in Mice

**Figure 6:**
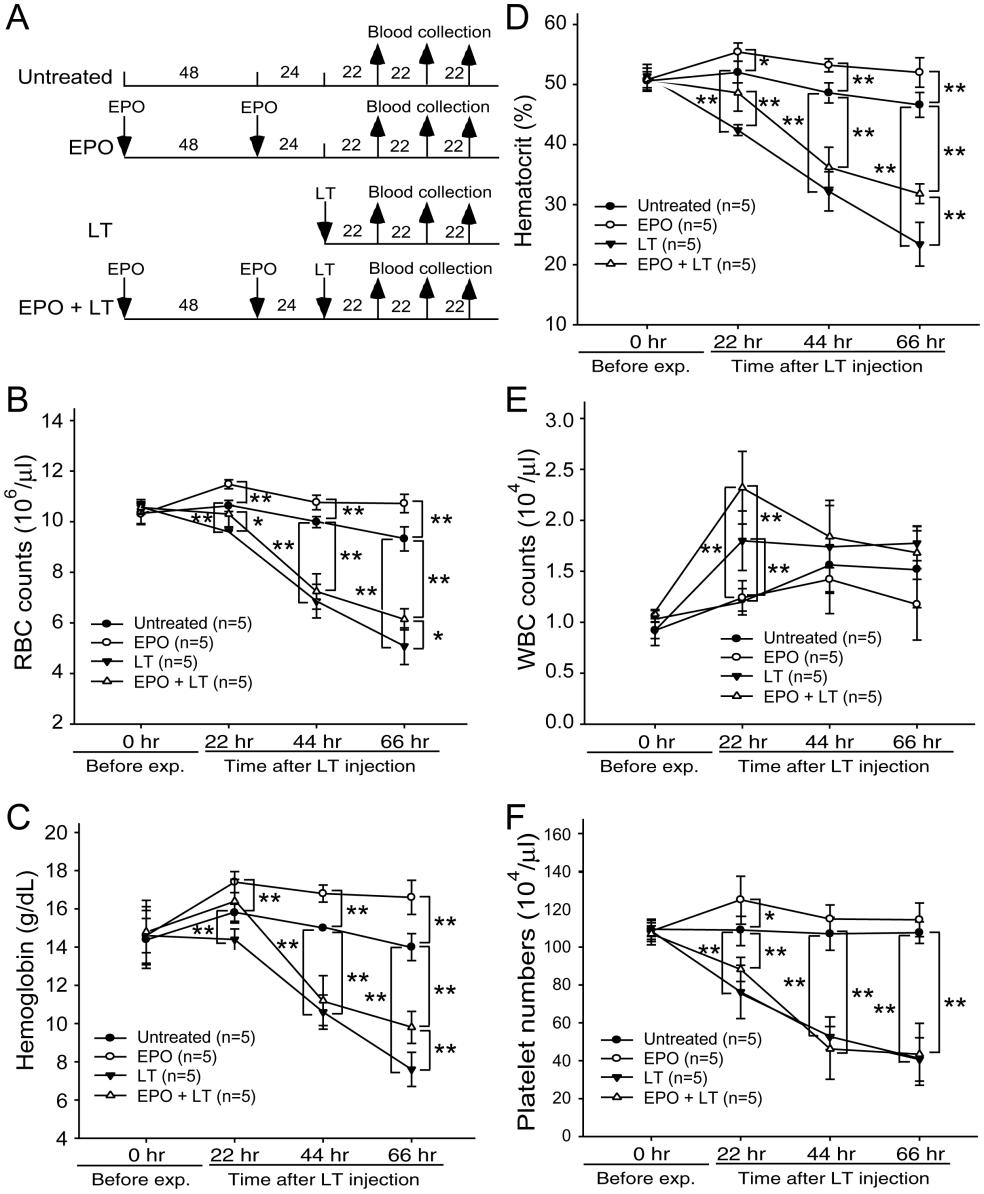
Reduced LT-mediated mortality using EPO treatments is associated with ameliorated anemic response. The experimental outline is illustrated in (A). The RBC counts (B), hemoglobin (C), hematocrit (D), WBC counts (E), and platelet counts (F) of mice treated with either EPO, LT, or EPO in addition LT at 22, 44, and 66 hours after LT treatments are shown. Untreated mice were used as negative controls. *p<0.05, ** *p*<0.01, comparisons between groups are indicated. Data are reported as mean ± SD, and represent results from 2 independent experiments.

**Figure 7:**
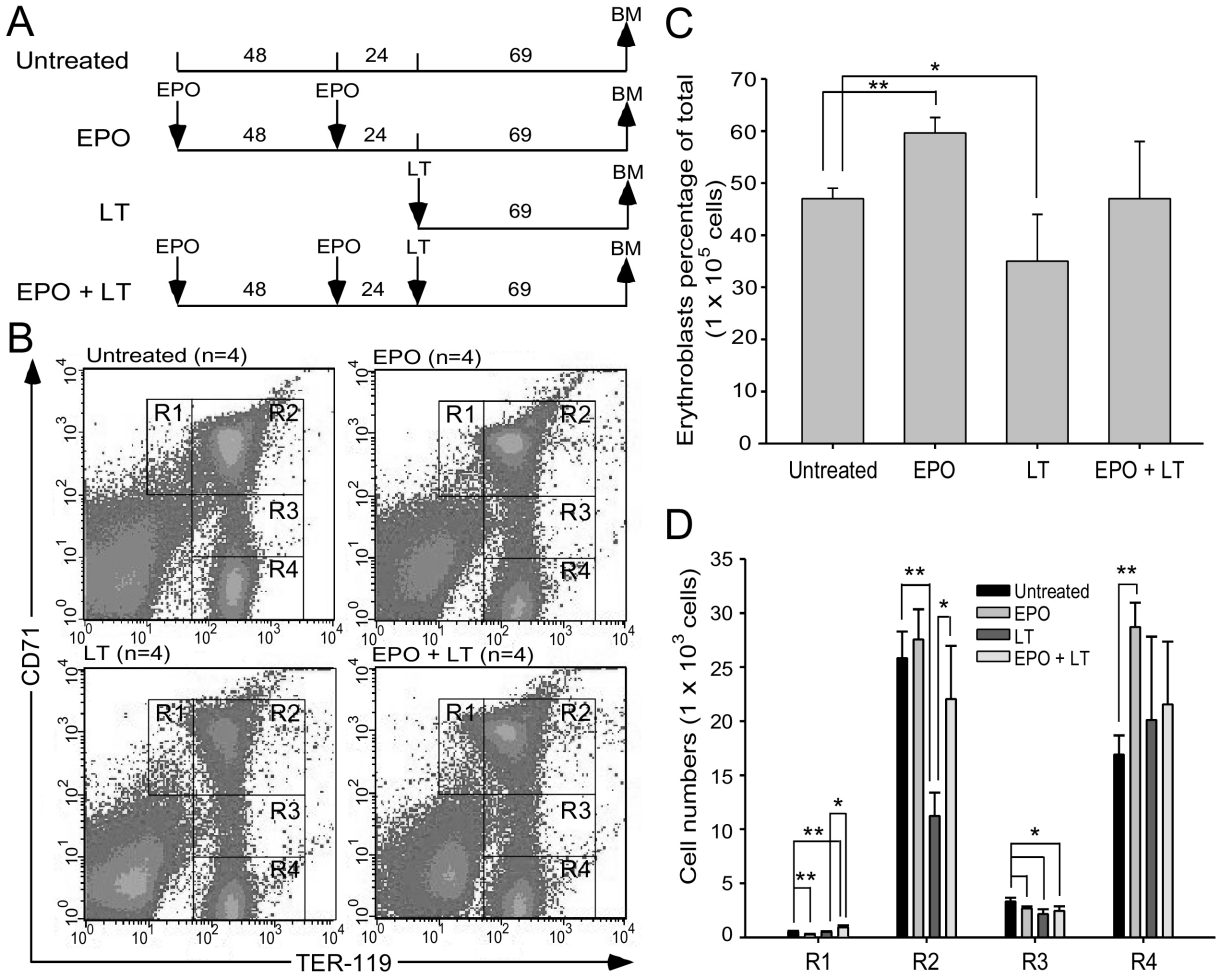
Beneficial effects of EPO treatments on survival are associated with rescued bone-marrow erythroblasts of LT-treated mice. The experimental outline is illustrated in (A). Flow cytometry analyses of BM cells were performed at 69 hours post-LT treatments. Following a previously described method used to distinguish the maturation stages of RBC precursors [33], the erythroblasts were gated as R1 (CD71^high^, TER-119^med^), R2 (CD71^high^, TER-119^high^), R3 (CD71^med^, TER-119^high^), and R4 (CD71^low^, TER-119^high^) in all groups (B). The relative cell population (% of total 1×10^5^) (C) and the cell numbers of all erythroid precursor cells (R1, R2, R3, and R4) in the respective groups are shown (D). *p<0.05, ** *p*<0.01 comparisons between groups are indicated. Data are reported as mean ± SD.

Upregulated plasma EPO levels were previously shown to coincide with hypoxic tissue damages in experimental mice [15]. EPO is a 30,400 dalton glycoprotein that positively regulates the BM erythroid cell proliferation, differentiation, and survival [30]. To investigate whether the induction of EPO was only a consequence of an acute phase response or a physiological counteraction to overcome LT-mediated erythrocyte suppression, recombinant EPO was administrated 1–3 days prior to LT treatments (Figure 5A). If LT-mediated hemolysis anemia and erythropoiesis suppression are associated with LT-mediated pathogenic progression, then increasing erythropoiesis using EPO treatments may have ameliorative effects. We found that a single injection of a lethal dose of LT (1.5 mg/kg, LF : PA = 1∶ 5) in mice resulted in 100% mortality (6/6 deaths) within 130 hours (Figure 5B). In contrast, the same dosage of LT induced only 50% mortality (3/6) in EPO-treated mice within 130 hours (Figure 5B; statistically significant, *p* = 0.029). Our previous study has found that LT treatments also induce thrombocytopenia and megakaryopoiesis suppression. These defects play certain roles in LT-induced lethal pathogenesis [31]. In addition, treatments of TPO can reduce LT-mediated mortality from 95.8% to 46.7% in mice [31]. This evidence prompted us to test whether combined treatments using EPO and TPO could achieve further amelioration on the lethal pathogenesis. Our data revealed that pretreatments using EPO and TPO could exert a complete (100%) rescue of the mice from LT-induced lethality (Figure 5B, TPO+EPO+LT groups). All surviving mice remained alive for up to 2 months (authors’ unpublished data). To verify whether reduced mortality caused by EPO pretreatments is associated with ameliorated suppression on erythropoiesis, a BFU-E colony assay was performed using BM cells from LT- and/or EPO-treated mice (Figure 5C, experimental outline). Analysis data revealed that the colony formation of BM-derived BFU-E was suppressed in LT-treated groups, while such suppression is significantly ameliorated by EPO pretreatments (Figure 5D, LT vs. EPO+LT groups). RBC counts, hemoglobin, and hematocrits can help diagnose anemia [32]. These parameters were measured to determine whether EPO rescue is associated with ameliorated anemia. Experimental mice were treated with or without LT and/or EPO, and the respective hematopoietic parameters such as RBC counts, hemoglobin, hematocrits, white blood cell (WBC) counts, and platelet counts were measured at various times for comparison (Figure 6A, experimental outline). The data revealed that all of these parameters except the WBC counts were reduced after LT treatments (Figure 6B–6F, untreated vs. LT groups), whereas EPO significantly ameliorated all LT-mediated suppressions except the low platelet counts (Figure 6B–6D and 6F, LT vs. EPO+LT groups). To verify whether the ameliorative effect of EPO on anemia can be attributed to an increase in erythropoiesis, BM cells of mice were isolated from femurs and tibiae at 69 hours post-LT treatments (Figure 7A, experimental outline). The surface expression of CD71 and TER-119 was measured to determine the maturation status of erythrocytes by using flow cytometry [33], [34]. CD71 is expressed in a wide variety of hematopoietic cells, including BFU-Es, CFU-Es, and proerythroblasts [27], whereas TER-119 is a late erythroid marker expressed on erythroblast cells [35]. These 2 markers allow researchers to distinguish among erythroid cell populations at 4 differentiation states: CD71^high^TER-119^med^ (Figure 7B, Region 1, R1), CD71^high^TER-119^high^ (Figure 7B, Region 2, R2), CD71^med^TER-119^high^ (Figure 7B, Region 3, R3), and CD71^low^TER-119^high^ (Figure 7B, Region 4, R4) [36]. Analysis data revealed that the percentage of total erythroblasts was reduced after LT treatment (Figure 7B, R1-to-R4 regions, and Figure 7C, untreated vs. LT), especially in the R2 and R3 populations (Figure 7D, R2 and R3 groups, untreated vs. LT) in BM. EPO pretreatment significantly increased the cell numbers in the R1 and R2 regions (Figure 7D, LT vs. EPO+LT). These results further suggest that LT suppresses erythropoiesis in mouse BM, and that EPO treatments can ameliorate this suppression (Figure 7B–7D).

## Discussion

Hemoglobin in RBCs is the principle molecule that serves as an oxygen carrier [37]. Suppressions of RBC counts, hemoglobin, and hematocrits by LT treatments (Figure 6B–6D) may theoretically contribute to the systemic hypoxia observed in LT-treated mice [15] and anthrax patients [16], [18]. LT-induced hemolysis is one of the potential causes of low RBC counts. However, the basal level of cell-free hemoglobin detected in mice plasma cannot fully explain the drastic drops in RBC counts and hematocrits (Figure 1D, 6B, and 6D). Because approximately 2.5 days (or 60 hours) are required to accomplish erythropoiesis in murine BM [38], LT-mediated suppression on erythropoiesis can theoretically be achieved before lethality occurs (Figure 5B, 78.5–126.5 hours).

The elicitation of apoptosis of primary CD34^+^-derived erythroblasts during *in vitro* differentiation (Figure 2, 3, 4) suggests that the suppressed erythropoiesis in BM may be caused by cell death of CD71^high^TER-119^high^ (R2) and CD71^med^TER-119^high^ (R3) populations (Figure 7B and 7D). The suppressive effect of LT on erythropoiesis also occurs in the spleen (Figure S1), a minor erythropoiesis site that is primarily induced under stress [39], [40]. These occurrences are in agreement with our colony-forming unit cell assays (*in vitro*, Figure 2F; *in vivo*, Figure 5D) and a previous report [41].

Previous studies have indicated that PA binds to the anthrax toxin receptor ATR1 on all lineages of hematopoietic progenitors in the bone marrow, including the erythroid progenitor lineage [41], [42], and suppresses hematopoiesis [41]. However, the mechanism involving LT-induced erythropoiesis suppression is unclear. In our study, stroma cells were supplied in the expansion stage but not in the *in vitro* differentiation stages (Figure 2A and 3A). Thus, an indirect suppressive effect through LT-elicited stroma-derived cytokines, which was suggested to suppress hematopoiesis [41], is not likely to be involved. LT is an MAPK inhibitor and cleaves the N-terminal domain of MKKs/MEKs [8], [9], disrupting 3 MAPK pathways. The carboxy-terminal region at residues 686–690 (HEXXH) of LF contains a putative zinc-biding site for its metalloprotease activity [43]. We previously demonstrated that the mutant LF^E687A^-composed LT has a reduced cytotoxicity and catalytic property against MEK1 and MEK2 ([29] and authors’ unpublished results). Our data suggests that the catalytic activity of LT is required for the suppressive effect on erythropoiesis (Figure 3).

Two major methods for tackling anthrax are currently available: vaccination and antibiotics [44], [45]. Animal experiment results show that it takes approximately 6 or more PA immunization cycles to elicit a weak immune response against anthrax. However, the protective immunity does not usually last long (authors’ unpublished data). In addition, because the toxic effect of LT is sufficient for lethality, animals may eventually die even after aggressive antibiotic therapies to eliminate the bacteria [46]. These results indicate that a specific treatment against LT is required. In this study, we demonstrated that pretreatments of EPO could reduce mortality in LT-injected mice by ameliorating RBC counts, hemoglobin, hematocrits, and erythropoiesis in BM (Figure 5, 6, 7). However, EPO contains nonerythropoietic properties, including tissue protection resulting from its ability to bind to injury-induced lower-affinity EPO receptors (EPORs), distinct from the high-affinity EPORs involved in erythropoiesis [47], [48]. In addition to the erythropoietic induction, the tissue-protective property of EPO may be one of the possible mechanisms explaining the rescue of LT-challenged mice [47], [49]. Studies have indicated that low levels of EPO (picomolar) are sufficient for erythropoiesis induction, whereas much higher levels of EPO (nanomolar) are required for tissue protection [47], [48]. EPO has a half-life of approximately 2 hours in the blood circulation [50]. Accordingly, to exclude the possibility of tissue protection, mice were treated with rhEPO using doses that kinetically declined into a range of concentrations to enhance erythropoiesis rather than tissue protection before LT was administered (Figure S2) [47]. The rhEPO was almost completely removed from the blood circulation of the mice within 24 hours (Figure S2), at a much faster clearing rate than for humans [47]. These results suggest that tissue protection is not involved in the EPO rescue. Our previous study showed that treatments of TPO could ameliorate LT-mediated mortality in mice [31]. This evidence prompted us to test whether combined treatments using EPO and TPO could also achieve amelioration on the lethal pathogenesis. Our data reveal that pretreatments using EPO and TPO could exert a complete (100%) rescue of the mice from LT-induced lethality (Figure 5B, TPO+EPO+LT groups). These results indicate the combined treatments of EPO and TPO is much potent than the single treatment of TPO alone [31]. TPO contains erythropoiesis-enhancing properties in certain conditions [51], whereas treatments of TPO alone did not significantly resolve the anemic symptoms of LT-challenged mice [Figure 7D of the reference [31]], indicating that the erythropoiesis-enhancing property of TPO is not sufficient to protect against LT-induced erythropoiesis suppression. Nonetheless, these results suggest that the platelet and RBC are critical cell types for maintaining homeostasis in LT-challenged mice. EPO administration is the well known medical treatment to enhance erythropoiesis in renal anemia [52], anemia of prematurity [53], and anemia with non-myeloid malignancies [54]. TPO treatment has been discontinued due to thrombocytopenia elicited by autoantibodies against endogenous TPO [55]. For clinical view, combined administration of EPO and second-generation of thrombopoietic agents, such as romiplostim and eltrombopag, will be used as a potential approach to treat anthrax.

Evidence from other studies has revealed that once anthrax enters a bacteremia stage, it inevitably leads to death, *even* with aggressive antibiotic therapy that prevents bacterial growth [17]. This is caused by toxin-mediated damages that are accumulated in the body, although the exact mechanism remains unclear [56]. Our data collectively suggested that LT-mediated suppression on megakaryopoiesis and erythropoiesis is part of LT-mediated pathophysiology. Protection from such damage to hematopoietic progenitor cells using specific growth factors can significantly reduce LT-induced animal death. The findings we have documented provide a novel perspective for the potential development of feasible alternative and additional therapeutic strategies against anthrax.

## Materials and Methods

### Ethics Statement

Cord bloods and the umbilical cords from full-term deliveries were collected by Mennonite Christian Hospital, Hualien, Taiwan. Informed written consents were provided by participants and obtained using protocols approved by the Research Ethics Committee of Mennonite Christian Hospital (Approval ID: 09-12-046-ER and 09-12-047-ER). All human samples were anonymized. The research methods used on the experimental mice were in accordance with the national guidelines of Animal Protection Act (Taiwan) and approved by the Institutional Animal Care and Use Committee, Tzu Chi University (Approval ID: 95017; Project: Potential roles of *Bacillus anthracis* lethal toxin on erythroid cells).

### Toxins

LT was purified and obtained as previously described [57]. Doses of LT refer to 1∶5 amounts of LF and PA (i.e., 120 µg LT equals 20 µg LF plus 100 µg PA). Recombinant wild-type LT (rLT) is composed of recombinant PA (rPA) and wild-type LF (rLF), and catalytic mutant LT (rLT^E687A^) is composed recombinant PA (rPA) and E687A-mutant LF (rLF^E687A^) [29], respectively. These recombinant proteins were produced and purified from *Escherichia coli*/SG13009 as previously described [29], using His-tagged affinity chromatography according to the manufacturer’s instructions (Qiagen, Hilden, Germany).

### Hemoglobin Detection

C57BL/6J mice (males, 6–8 weeks old) were purchased from the National Laboratory Animal Center (Taipei, Taiwan) and maintained in pathogen-free conditions in the experimental animal center at Tzu Chi University. For the *in vitro* hemoglobin assay, peripheral blood collected from the retro-orbital plexi of C57BL/6J mice were mixed with anticoagulant acid citrate dextrose formula A (ACD-A : 38 mM citric acid, 75 mM trisodium citrate, 139 mM D-glucose, 12.5 mM EDTA) (blood : ACD-A = 8∶1) [58] and then incubated with or without LT (200 ng/ml) at 37°C for 2 hours [19]. Plasma hemoglobin was measured using Drabkin’s reagent (Sigma-Aldrich, St. Louis, MO, USA) by detecting the absorbance at 540 nm. The standard curve for hemoglobin was obtained by serial dilution of the whole blood from the peripheral blood of untreated mice, in which the hemoglobin concentration was determined using an automated hematology analyzer (KX-21, Sysmex Corporation, Kobe, Japan). Hemoglobin concentrations (µg/dL) of the samples were then obtained through comparisons of the standard curve. To determine LT-induced hemolysis *in vivo*, C57BL/6J mice were retro-orbitally injected with or without a lethal dose of LT (1.5 mg/kg in 250 µl saline). Untreated and saline-treated mice were used as negative controls. The level of plasma cell-free hemoglobin was measured at 22, 44, and 66 hours post-LT treatments using Drabkin’s reagent.

### Erythroid Colony-forming Cells Assay

Mouse (C57BL/6J) bone marrow (BM) cells from femurs and tibiae were collected by flushing using RPMI-1640 containing 20% anticoagulant ACD-A. The cell suspensions were passed through a 55 µm nylon mesh to remove cell aggregates. Red blood cells (RBCs) were depleted by adding a hypotonic buffer (153 mM NH_4_Cl and 17 mM Tris-HCl) at room temperature for 10 minutes. Cells (9×10^5^/ml, 100 µl) were then resuspended in Iscove’s Modified Dulbecco’s Medium (IMDM) (StemCell Technologies, Vancouver, BC, Canada) and mixed with a 1 ml semisolid methylcellulose-based medium containing 3 units of EPO (MethoCult-M3334, StemCell Technologies, Vancouver, BC, Canada). Each 1.1 ml of methylcellulose cell suspension was mixed with or without a varied LT dose (200 ng/ml or 20 ng/ml) and seeded in 35 mm dishes. Finally, the cultures were incubated at 37°C for either 7 or 14 days.

### Erythrocyte *in vitro* Culture and Flow Cytometry Assay

Mononuclear cells of human cord blood were purified using Ficoll-Paque Plus (GE Healthcare Bio-Sciences). Fresh CD34^+^ cells were purified using CD34-microbeads by a Miltenyi VarioMACS device (Miltenyi Biotec), according to the manufacturer’s instructions. To induce erythroid differentiation, isolated CD34^+^ cells were initially co-cultured with umbilical-cord mesenchymal stem cells (MSC, Wharton’s jelly [59]) in a cell-culture IMDM (Gibco) containing 10% fetal bovine serum (FBS, Biological Industries, Kibbutz Beit Haemek, Israel), 10 ng/ml recombinant human thrombopoietin (rhTPO, PeproTech), 20 ng/ml recombinant human interleukin-3 (rhIL-3, PeproTech), 30.5 ng/ml recombinant human stem cell factor (rhSCF, PeproTech), and 22.3 ng/ml recombinant human Flt-3 ligand (rhFLt3-L, PeproTech) for 4–5 days to expand the number of cells [60]. Approximately 5×10^5^ CD34^+^ cells were then cultured in 1 ml IMDM (Gibco) supplemented with 3% FBS, 200 µg/ml iron-saturated human transferrin (Sigma-Aldrich), 90 ng/ml ferric nitrate (Sigma-Aldrich), 50 µg/ml insulin, 10^−6^ M hydrocortisone (Sigma-Aldrich), 4 mM l-glutamine, 100 U/l penicillin, 100 mg/ml streptomycin and a cocktail of cytokines administered as follows: 5 ng/ml IL-3, 100 ng/ml SCF, and 3 IU/ml EPO were administered on Days 0–8; cells were diluted 2-fold on Day 4; and erythroid cells were cultured in the presence of 3 IU/ml EPO and MSC condition medium for further maturation on Days 8 to 16 [61]. On the differentiation days, Days 0, 4, 8, and 12, LT (20 ng/ml) was added. For the recombinant anthrax toxins, rPA (5 µg/ml), rLF (5 µg/ml), rLF^E687A^ (5 µg/ml), rLT (5 µg/ml rPA +5 µg/ml rLF), and rLT^E687A^ (5 µg/ml rPA +5 µg/ml rLF^E687A^) were added on Day 8. After 4 days, the total cells of the respective groups were isolated for flow cytometry analysis. Untreated, diluent-treated (glycerol:saline = 1∶1), and bovine serum albumin (BSA)-treated (10 µg/ml) groups were used as negative controls. The erythrocytic surface marker expressions were stained using anti-human CD71-APC (eBioscience) and anti-human CD235a-PE (eBioscience) antibodies. To analyze apoptotic cells, Annexin V-APC (BD Pharmingen) and anti-active caspase-3 antibodies conjugated with FITC (BD Pharmingen) were used, and then analyzed using flow cytometry and the CellQuest program (Becton-Dickinson).

### DNA Content Analysis

Cells were washed twice using phosphate buffered saline (PBS), fixed with ice-cold 70% ethanol in PBS at –20°C for 2 hours, and then washed with PBS and resuspended in 500 µl staining solution containing propidium iodine (PI) (20 µg/ml PI, 0.1% Triton X-100 and 0.2 mg/ml RNase A in PBS) and incubated at room temperature for 30 minutes. The DNA content of the cells was analyzed using a flow cytometer (FACSCalibur, Becton-Dickinson, San Jose, CA, USA).

### EPO Rescue Experiments (Mortality, Erythroid Colony-forming Cell Assay, Peripheral Blood Hematopoietic Parameters, and Bone Marrow Analysis)

C57BL/6J mice (males, 10–11 weeks old) were treated with recombinant human EPO (rhEPO, Neorecormon ®, Roche, Mannheim, Germany) (2 IU/g, in 250 µl saline) and/or murine TPO (CytoLab, Rehovot, Israel) (0.25 µg/mice, in 250 µl saline) twice by using a retro-orbital injection at 72 and 24 hours before administration of a lethal dose of LT (1.5 mg/kg in 250 µl saline, using retro-orbital injection, with approximately 95.8% mortality [31]). Untreated and EPO-treated groups without further LT treatments served as controls. As there is no suitable parameter to predict the death/survival for LT-challenged mice, we use death as an endpoint. The mortality and survival duration of the mice were recorded after LT treatments. The experimental mice were monitored every 4–6 hours. The surveillance showed that LT-challenges reduce behavioral activities of mice before death without body weight loss and any obvious discomfort (such as abnormal behavior and convulsions). Surviving mice were sacrificed by cervical dislocation 69 hours after LT treatments. The BM cells were collected and performed *in vivo* in the erythroid colony-forming cell assay (Materials and Methods: **Erythroid colony-forming cell assay**). To measure the hematological parameters [RBC counts, hemoglobin level, white blood cell counts, hematocrits (Hcts), and platelet counts], blood samples were collected 22, 44, and 66 hours from the retro-orbital plexi of the mice after LT treatments. Collected blood was mixed with the anticoagulant ACD-A (blood: ACD-A = 1∶9). The hematological parameters were determined using an automated hematology analyzer (KX-21, Sysmex Corporation, Kobe, Japan). To detect erythroblast-specific surface markers, BM cells of mice were collected from femurs and tibiae 69 hours after LT treatments. The femurs and tibiae were flushed using the RPMI-1640 medium supplemented with 20% anticoagulant ACD-A through 30G needles. The cell suspensions were passed through a 55 µm nylon mesh to remove the cell aggregates. Cells were blocked with 5% BSA in an RPMI medium at 37°C for 1 hour and then incubated in a 500 µl RPMI-1640 medium with 1 µl of an FITC-conjugated rat anti-mouse CD71 antibody (BioLegend) and 3 µl of R-Phycoerythrin (R-PE)-conjugated rat anti-mouse TER-119 antibody (BD Immunocytometry System) at 37°C for 1 hour. After PBS washes, the cells were analyzed using a FACSCalibur flow cytometer and CellQuest™ Pro program (Becton-Dickinson, San Jose, CA, USA).

### Statistics

Means, standard deviations (SDs), and statistics for quantifiable data were calculated using Microsoft Office Excel 2003 for Windows. Comparisons between groups were made using the two-tailed Student *t* test. A *p* value of <0.05 was considered significant.

## Supporting Information

Figure S1 **LT-mediated mortality is associated with reduced number of erythroblast cells in spleen.** The experimental outline of spleen experiments is illustrated (A). Flow cytometry analysis of spleen cells were performed at 69 hours after LT treatments. To determine the maturation stages of RBC precursors, the erythroblast cells were gated as R1 (CD71^high^, TER-119^med^), R2 (CD71^high^, TER-119^high^), R3 (CD71^med^, TER-119^high^), and R4 (CD71^low^, TER-119^high^) in all groups (B). The relative cell population (% of total 1×10^5^) (C) and the cell numbers of all erythroblast cells (R1, R2, R3, and R4) in respective groups are showed (D). *p<0.05, ** *p*<0.01, comparisons between groups are indicated. Data are reported as mean ± standard deviation (SD).
(TIF)

Figure S2 **Plasma EPO concentration after EPO injection.** The experimental outline of EPO immunoassay is showed (A). EPO concentration of plasma after two sequential EPO injections was examined 30 minutes and 24 hours after the second EPO injection by ELISA (B). Data are reported as mean ± standard deviation (SD) except the 30-minute group showed as a mean value. EPO concentration was calculated according to a standard curve showed on (C).
(TIF)

Methods S1 **Supplemental experimental procedures.**
(PDF)

## References

[1] Mock M, Fouet A (2001) Anthrax. Annu Rev Microbiol 55: 647–671. 11544370 10.1146/annurev.micro.55.1.647

[2] Abramova FA, Grinberg LM, Yampolskaya OV, Walker DH (1993) Pathology of inhalational anthrax in 42 cases from the Sverdlovsk outbreak of 1979. Proc Natl Acad Sci U S A 90: 2291–2294. 8460135 10.1073/pnas.90.6.2291 PMC46072

[3] Dixon TC, Meselson M, Guillemin J, Hanna PC (1999) Anthrax. N Engl J Med 341: 815–826. 10477781 10.1056/NEJM199909093411107

[4] Shafazand S, Doyle R, Ruoss S, Weinacker A, Raffin TA (1999) Inhalational anthrax: epidemiology, diagnosis, and management. Chest 116: 1369–1376. 10559102 10.1378/chest.116.5.1369

[5] Brossier F, Mock M (2001) Toxins of Bacillus anthracis. Toxicon 39: 1747–1755. 11595637 10.1016/s0041-0101(01)00161-1

[6] Collier RJ, Young JA (2003) Anthrax toxin. Annu Rev Cell Dev Biol 19: 45–70. 14570563 10.1146/annurev.cellbio.19.111301.140655

[7] Mourez M (2004) Anthrax toxins. Rev Physiol Biochem Pharmacol 152: 135–164. 15549606 10.1007/s10254-004-0028-2

[8] Bardwell AJ, Abdollahi M, Bardwell L (2004) Anthrax lethal factor-cleavage products of MAPK (mitogen-activated protein kinase) kinases exhibit reduced binding to their cognate MAPKs. Biochem J 378: 569–577. 14616089 10.1042/BJ20031382 PMC1223970

[9] Turk BE (2007) Manipulation of host signalling pathways by anthrax toxins. Biochem J 402: 405–417. 17313374 10.1042/BJ20061891

[10] Hagemann C, Blank JL (2001) The ups and downs of MEK kinase interactions. Cell Signal 13: 863–875. 11728826 10.1016/s0898-6568(01)00220-0

[11] Wada T, Penninger JM (2004) Mitogen-activated protein kinases in apoptosis regulation. Oncogene 23: 2838–2849. 15077147 10.1038/sj.onc.1207556

[12] Bradley KA, Mogridge J, Mourez M, Collier RJ, Young JA (2001) Identification of the cellular receptor for anthrax toxin. Nature 414: 225–229. 11700562 10.1038/n35101999

[13] Moayeri M, Leppla SH (2004) The roles of anthrax toxin in pathogenesis. Curr Opin Microbiol 7: 19–24. 15036135 10.1016/j.mib.2003.12.001

[14] Cui X, Moayeri M, Li Y, Li X, Haley M, et al (2004) Lethality during continuous anthrax lethal toxin infusion is associated with circulatory shock but not inflammatory cytokine or nitric oxide release in rats. Am J Physiol Regul Integr Comp Physiol 286: R699–709. 14715494 10.1152/ajpregu.00593.2003

[15] Moayeri M, Haines D, Young HA, Leppla SH (2003) Bacillus anthracis lethal toxin induces TNF-alpha-independent hypoxia-mediated toxicity in mice. J Clin Invest 112: 670–682. 12952916 10.1172/JCI17991 PMC182199

[16] Freedman A, Afonja O, Chang MW, Mostashari F, Blaser M, et al (2002) Cutaneous anthrax associated with microangiopathic hemolytic anemia and coagulopathy in a 7-month-old infant. JAMA 287: 869–874. 11851579 10.1001/jama.287.7.869

[17] Jernigan JA, Stephens DS, Ashford DA, Omenaca C, Topiel MS, et al (2001) Bioterrorism-related inhalational anthrax: the first 10 cases reported in the United States. Emerg Infect Dis 7: 933–944. 11747719 10.3201/eid0706.010604 PMC2631903

[18] Mina B, Dym JP, Kuepper F, Tso R, Arrastia C, et al (2002) Fatal inhalational anthrax with unknown source of exposure in a 61-year-old woman in New York City. JAMA 287: 858–862. 11851577 10.1001/jama.287.7.858

[19] Wu AG, Alibek D, Li YL, Bradburne C, Bailey CL, et al (2003) Anthrax toxin induces hemolysis: an indirect effect through polymorphonuclear cells. J Infect Dis 188: 1138–1141. 14551883 10.1086/378516

[20] Huo XF, Zhang JW (2005) Annexin1 regulates the erythroid differentiation through ERK signaling pathway. Biochem Biophys Res Commun 331: 1346–1352. 15883023 10.1016/j.bbrc.2005.04.049

[21] Zhang J, Lodish HF (2004) Constitutive activation of the MEK/ERK pathway mediates all effects of oncogenic H-ras expression in primary erythroid progenitors. Blood 104: 1679–1687. 15166036 10.1182/blood-2004-04-1362

[22] Jacobs-Helber SM, Sawyer ST (2004) Jun N-terminal kinase promotes proliferation of immature erythroid cells and erythropoietin-dependent cell lines. Blood 104: 696–703. 15059850 10.1182/blood-2003-05-1754

[23] Moosavi MA, Yazdanparast R, Lotfi A (2007) ERK1/2 inactivation and p38 MAPK-dependent caspase activation during guanosine 5′-triphosphate-mediated terminal erythroid differentiation of K562 cells. Int J Biochem Cell Biol 39: 1685–1697. 17543571 10.1016/j.biocel.2007.04.016

[24] Kristiansen M, Graversen JH, Jacobsen C, Sonne O, Hoffman HJ, et al (2001) Identification of the haemoglobin scavenger receptor. Nature 409: 198–201. 11196644 10.1038/35051594

[25] Nagel RL, Gibson QH (1971) The binding of hemoglobin to haptoglobin and its relation to subunit dissociation of hemoglobin. J Biol Chem 246: 69–73. 5541775

[26] Rother RP, Bell L, Hillmen P, Gladwin MT (2005) The clinical sequelae of intravascular hemolysis and extracellular plasma hemoglobin: a novel mechanism of human disease. JAMA 293: 1653–1662. 15811985 10.1001/jama.293.13.1653

[27] Trowbridge IS, Lesley J, Schulte R (1982) Murine cell surface transferrin receptor: studies with an anti-receptor monoclonal antibody. J Cell Physiol 112: 403–410. 6290505 10.1002/jcp.1041120314

[28] Gahmberg CG, Jokinen M, Andersson LC (1978) Expression of the major sialoglycoprotein (glycophorin) on erythroid cells in human bone marrow. Blood 52: 379–387. 667363

[29] Chang HH, Tsai MF, Chung CP, Chen PK, Hu HI, et al (2006) Single-step purification of recombinant anthrax lethal factor from periplasm of Escherichia coli. J Biotechnol 126: 277–285. 16797097 10.1016/j.jbiotec.2006.04.038

[30] Fisher JW (2003) Erythropoietin: physiology and pharmacology update. Exp Biol Med (Maywood) 228: 1–14. 12524467 10.1177/153537020322800101

[31] Chen PK, Chang HH, Lin GL, Wang TP, Lai YL, et al.. (2013) Suppressive effects of anthrax lethal toxin on megakaryopoiesis. PLoS ONE 8 e59512. 10.1371/journal.pone.0059512 PMC3605335 23555687

[32] George-Gay B, Parker K (2003) Understanding the complete blood count with differential. J Perianesth Nurs 18: 96–114; quiz 115–117. 10.1053/jpan.2003.50013 12710004

[33] Socolovsky M, Nam H, Fleming MD, Haase VH, Brugnara C, et al (2001) Ineffective erythropoiesis in Stat5a(−/−)5b(−/−) mice due to decreased survival of early erythroblasts. Blood 98: 3261–3273. 11719363 10.1182/blood.v98.12.3261

[34] Zhang J, Socolovsky M, Gross AW, Lodish HF (2003) Role of Ras signaling in erythroid differentiation of mouse fetal liver cells: functional analysis by a flow cytometry-based novel culture system. Blood 102: 3938–3946. 12907435 10.1182/blood-2003-05-1479

[35] Kina T, Ikuta K, Takayama E, Wada K, Majumdar AS, et al (2000) The monoclonal antibody TER-119 recognizes a molecule associated with glycophorin A and specifically marks the late stages of murine erythroid lineage. Br J Haematol 109: 280–287. 10848813 10.1046/j.1365-2141.2000.02037.x

[36] Halupa A, Bailey ML, Huang K, Iscove NN, Levy DE, et al (2005) A novel role for STAT1 in regulating murine erythropoiesis: deletion of STAT1 results in overall reduction of erythroid progenitors and alters their distribution. Blood 105: 552–561. 15213094 10.1182/blood-2003-09-3237

[37] Inayat MS, Bernard AC, Gallicchio VS, Garvy BA, Elford HL, et al (2006) Oxygen carriers: a selected review. Transfus Apher Sci 34: 25–32. 16376617 10.1016/j.transci.2005.09.005

[38] Mary JY, Valleron AJ, Croizat H, Frindel E (1980) Mathematical analysis of bone marrow erythropoiesis: application to C3H mouse data. Blood Cells 6: 241–262. 7378594

[39] Mide SM, Huygens P, Bozzini CE, Fernandez Pol JA (2001) Effects of human recombinant erythropoietin on differentiation and distribution of erythroid progenitor cells on murine medullary and splenic erythropoiesis during hypoxia and post-hypoxia. In Vivo 15: 125–132. 11317516

[40] Vacha J, Hola J, Dungel J, Znojil V (1982) The distribution of erythropoiesis over the various anatomical regions of the erythropoietic system in some inbred strains of mice. Exp Hematol 10: 768–773. 7173343

[41] Rameshwar P, Wong EW, Connell ND (2012) Effects by anthrax toxins on hematopoiesis: a key role for cytokines as mediators. Cytokine 57: 143–149. 22082805 10.1016/j.cyto.2011.10.013

[42] Liu K, Wong EW, Schutzer SE, Connell ND, Upadhyay A, et al (2009) Non-canonical effects of anthrax toxins on haematopoiesis: implications for vaccine development. J Cell Mol Med 13: 1907–1919. 18752638 10.1111/j.1582-4934.2008.00477.x PMC2819559

[43] Klimpel KR, Arora N, Leppla SH (1994) Anthrax toxin lethal factor contains a zinc metalloprotease consensus sequence which is required for lethal toxin activity. Mol Microbiol 13: 1093–1100. 7854123 10.1111/j.1365-2958.1994.tb00500.x

[44] Holty JE, Bravata DM, Liu H, Olshen RA, McDonald KM, et al (2006) Systematic review: a century of inhalational anthrax cases from 1900 to 2005. Ann Intern Med 144: 270–280. 16490913 10.7326/0003-4819-144-4-200602210-00009

[45] Tournier JN, Ulrich RG, Quesnel-Hellmann A, Mohamadzadeh M, Stiles BG (2009) Anthrax, toxins and vaccines: a 125-year journey targeting Bacillus anthracis. Expert Rev Anti Infect Ther 7: 219–236. 19254170 10.1586/14787210.7.2.219

[46] Shoop WL, Xiong Y, Wiltsie J, Woods A, Guo J, et al (2005) Anthrax lethal factor inhibition. Proc Natl Acad Sci U S A 102: 7958–7963. 15911756 10.1073/pnas.0502159102 PMC1138260

[47] Brines M, Cerami A (2008) Erythropoietin-mediated tissue protection: reducing collateral damage from the primary injury response. J Intern Med 264: 405–432. 19017170 10.1111/j.1365-2796.2008.02024.x

[48] Hand CC, Brines M (2011) Promises and pitfalls in erythopoietin-mediated tissue protection: are nonerythropoietic derivatives a way forward? J Investig Med 59: 1073–1082. 10.231/JIM.0b013e3181ed30bf PMC3023830 20683348

[49] Chateauvieux S, Grigorakaki C, Morceau F, Dicato M, Diederich M (2011) Erythropoietin, erythropoiesis and beyond. Biochem Pharmacol 82: 1291–1303. 21782802 10.1016/j.bcp.2011.06.045

[50] Fukuda MN, Sasaki H, Lopez L, Fukuda M (1989) Survival of recombinant erythropoietin in the circulation: the role of carbohydrates. Blood 73: 84–89. 2910371

[51] Ratajczak MZ, Ratajczak J, Marlicz W, Pletcher CH Jr, Machalinski B, et al (1997) Recombinant human thrombopoietin (TPO) stimulates erythropoiesis by inhibiting erythroid progenitor cell apoptosis. Br J Haematol 98: 8–17. 9233556 10.1046/j.1365-2141.1997.1802997.x

[52] de Francisco AL, Pinera C (2011) Anemia trials in CKD and clinical practice: refining the approach to erythropoiesis-stimulating agents. Contrib Nephrol 171: 248–254. 21625120 10.1159/000327173

[53] Gallagher PG, Ehrenkranz RA (1993) Erythropoietin therapy for anemia of prematurity. Clin Perinatol 20: 169–191. 8458164

[54] Shehata N, Walker I, Meyer R, Haynes AE, Imrie K, et al (2008) The use of erythropoiesis-stimulating agents in patients with non-myeloid hematological malignancies: a systematic review. Ann Hematol 87: 961–973. 18629499 10.1007/s00277-008-0525-5

[55] Li J, Yang C, Xia Y, Bertino A, Glaspy J, et al (2001) Thrombocytopenia caused by the development of antibodies to thrombopoietin. Blood 98: 3241–3248. 11719360 10.1182/blood.v98.12.3241

[56] Rainey GJ, Young JA (2004) Antitoxins: novel strategies to target agents of bioterrorism. Nat Rev Microbiol 2: 721–726. 15372082 10.1038/nrmicro977

[57] Kau JH, Lin CG, Huang HH, Hsu HL, Chen KC, et al (2002) Calyculin A sensitive protein phosphatase is required for Bacillus anthracis lethal toxin induced cytotoxicity. Curr Microbiol 44: 106–111. 11815854 10.1007/s00284-001-0059-8

[58] Brown AS, Hong Y, de Belder A, Beacon H, Beeso J, et al (1997) Megakaryocyte ploidy and platelet changes in human diabetes and atherosclerosis. Arterioscler Thromb Vasc Biol 17: 802–807. 9108797 10.1161/01.atv.17.4.802

[59] Bakhshi T, Zabriskie RC, Bodie S, Kidd S, Ramin S, et al (2008) Mesenchymal stem cells from the Wharton’s jelly of umbilical cord segments provide stromal support for the maintenance of cord blood hematopoietic stem cells during long-term ex vivo culture. Transfusion 48: 2638–2644. 18798803 10.1111/j.1537-2995.2008.01926.x PMC3444149

[60] Yao CL, Feng YH, Lin XZ, Chu IM, Hsieh TB, et al (2006) Characterization of serum-free ex vivo-expanded hematopoietic stem cells derived from human umbilical cord blood CD133(+) cells. Stem Cells Dev 15: 70–78. 16522164 10.1089/scd.2006.15.70

[61] Baek EJ, Kim HS, Kim S, Jin H, Choi TY, et al (2008) In vitro clinical-grade generation of red blood cells from human umbilical cord blood CD34+ cells. Transfusion 48: 2235–2245. 18673341 10.1111/j.1537-2995.2008.01828.x

