# Supplementary figures and images for: Erythropoiesis Suppression Is Associated with Anthrax Lethal Toxin-Mediated Pathogenic Progression
Source: PLoS One. 2013 Aug 19;8(8):e71718. doi: 10.1371/journal.pone.0071718 (PMC3747219; doi:10.1371/journal.pone.0071718)

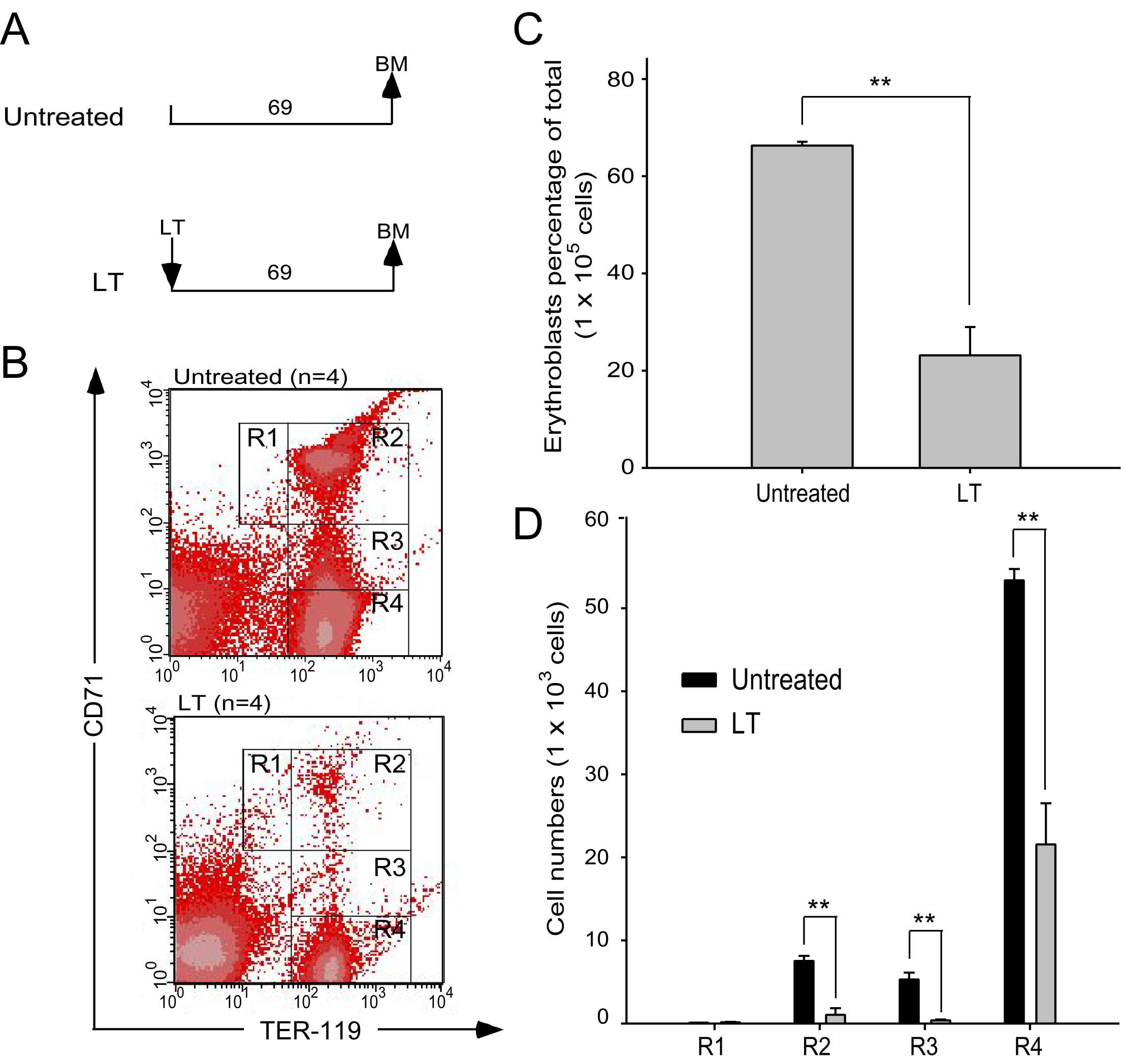

Supplement: Figure S1 — LT-mediated mortality is associated with reduced number of erythroblast cells in spleen. The experimental outline of spleen experiments is illustrated (A). Flow cytometry analysis of spleen cells were performed at 69 hours after LT treatments. To determine the maturation stages of RBC precursors, the erythroblast cells were gated as R1 (CD71high, TER-119med), R2 (CD71high, TER-119high), R3 (CD71med, TER-119high), and R4 (CD71low, TER-119high) in all groups (B). The relative cell population (% of total 1×10^5^) (C) and the cell numbers of all erythroblast cells (R1, R2, R3, and R4) in respective groups are showed (D). *p<0.05, ** p<0.01, comparisons between groups are indicated. Data are reported as mean ± standard deviation (SD). (TIF) [file pone.0071718.s001.tif]

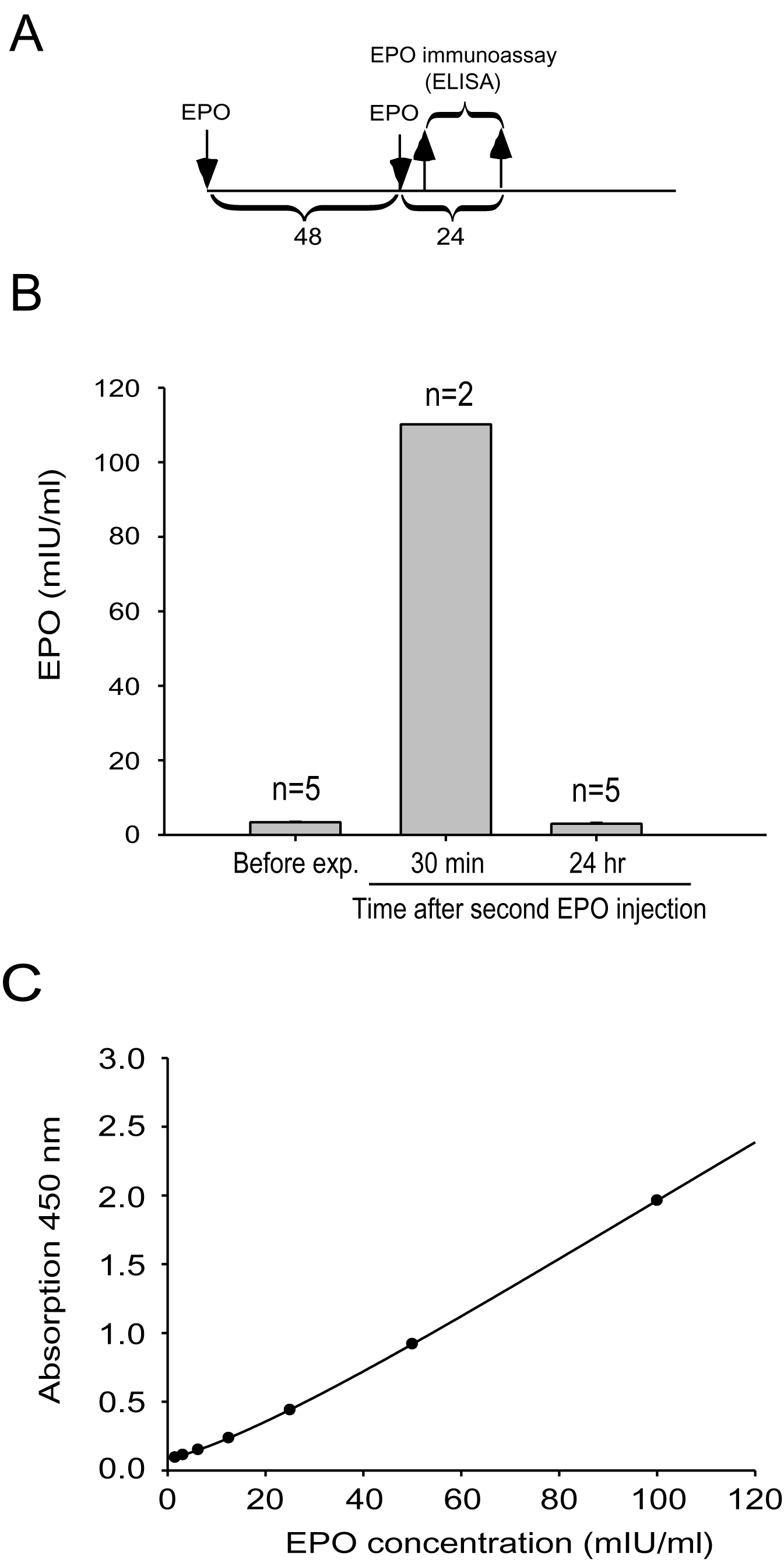

Supplement: Figure S2 — Plasma EPO concentration after EPO injection. The experimental outline of EPO immunoassay is showed (A). EPO concentration of plasma after two sequential EPO injections was examined 30 minutes and 24 hours after the second EPO injection by ELISA (B). Data are reported as mean ± standard deviation (SD) except the 30-minute group showed as a mean value. EPO concentration was calculated according to a standard curve showed on (C). (TIF) [file pone.0071718.s002.tif]
