# Supplementary material for: Erythropoiesis Suppression Is Associated with Anthrax Lethal Toxin-Mediated Pathogenic Progression
Source: PLoS One. 2013 Aug 19;8(8):e71718. doi: 10.1371/journal.pone.0071718 (PMC3747219; doi:10.1371/journal.pone.0071718)
Supplement: Methods S1 — Supplemental experimental procedures. (PDF) [file pone.0071718.s003.pdf]

Supplemental experimental procedures

Spleen erythropoiesis analysis

C57BL/6J mice (males, 6-8 weeks old) were injected with a lethal dose of LT (1.5 mg/kg, in 250 μl saline, retro-orbital injection). Untreated groups without LT administrations were served as negative controls. After 69 hours LT treatments, mouse splenocytes were collected through flushing spleens with RPMI-1640 medium supplemented with 20% anticoagulant acid citrate dextrose, formula A (ACD-A) by 30-gauge needles. The cell suspensions were passed through a 55 μm nylon mesh to remove cell aggregates. Cells were blocked with 5% BSA in RPMI medium at 37°C for 1 hour and then incubated in 500 μl RPMI-1640 medium with 1 μl of FITC-conjugated rat anti-mouse CD71 antibody (BioLegend) and 3 μl of R-Phycoerythrin (R-PE)-conjugated rat anti-mouse TER-119 antibody (BD Immunocytometry System) at 37°C for 1 hour. After washing with PBS, the cells were then analyzed by a FACSCalibur flow cytometer and the CellQuest™ Pro program (Becton-Dickinson, San Jose, CA, USA).

EPO immunoassay

C57BL/6J mice (males, 6-7 weeks old) were injected with recombinant human EPO (rhEPO, Neorecormon®, Roche, Mannheim, Germany) (2 IU/g, in 250 μl saline) twice through retro-orbital route at 72 and 24 hours before EPO immunoassay. Plasma EPO levels were determined by Human Erythropoietin Platinum enzyme-linked immunosorbent assays (ELISA) kit (eBioScience, San Diego, CA).
